# From Laparoscopic TAPP to l-GPSpX: A Pilot Reclassification of Hernia Surgery Acronyms Using the Muysoms Nomenclature

**DOI:** 10.3389/jaws.2025.15705

**Published:** 2025-11-07

**Authors:** Francesco Brucchi, Gianlorenzo Dionigi

**Affiliations:** 1 University of Milan, Milan, Italy; 2 Division of General and Endocrine Surgery, Istituto Auxologico Italiano, IRCCS (Istituto di Ricovero e Cura a Carattere Scientifco), Milan, Italy; 3 Department of Pathophysiology and Transplantation, University of Milan, Milan, Italy

**Keywords:** hernia, classification, acronyms, abdominal wall surgery, surgical taxonomy

Dear Editor,

The terminology of abdominal wall surgery has long been dominated by historical acronyms such as TAPP, TEP, IPOM, or MILOS. While widely adopted, these acronyms often fail to convey critical information regarding mesh type, fixation, or precise anatomical plane. In addition, the same surgical technique is frequently described by two or more different acronyms, creating unnecessary heterogeneity. This ambiguity not only confuses readers but also complicates evidence synthesis, hampering meta-analyses and guideline development by making the retrieval and classification of studies more difficult. The recently proposed Muysoms nomenclature addresses these issues by providing a rationalized framework that codes each procedure through five elements: approach, hernia type, mesh position, mesh type, and fixation [1].

To explore its feasibility, we retrospectively applied this system to a pilot sample of 13 published studies (16 treatment arms) covering groin, ventral, and robotic approaches [2–14]. Each technique was reclassified from its traditional acronym into the new format (e.g., *TAPP → l-GPSpX*).

Our analysis revealed:

- Approach and hernia type were always identifiable (100%) once the full text was reviewed, but remained ambiguous when relying on acronyms alone. For instance, *eTEP* could indicate ventral, incisional, or umbilical repairs. Moreover, the same technique was sometimes described using two or more different acronyms (TARUP aka TARM), further increasing heterogeneity.
- Mesh position was consistently inferred from the acronym (e.g., TAPP = preperitoneal, IPOM = intraperitoneal).
- Mesh type and fixation were rarely specified by acronyms, and required careful reading of the methods or looking at the figures; they remained missing in 19% and 25% of cases, respectively.

These findings highlight both the limitations of legacy acronyms and the added value of the Muysoms nomenclature, which captures essential technical features in a standardized, machine- and human-readable format. Importantly, the system allows retroactive mapping of historical literature, paving the way for harmonized meta-analyses and registry integration.

Furthermore, beyond our pilot findings, the value of this system lies in its conceptual design. As highlighted by Muysoms et al., more than one hundred acronyms have been introduced in hernia surgery, many inconsistently applied to the same techniques. By replacing acronym proliferation with a modular combination of five elements, the Muysoms nomenclature offers a future-proof, unambiguous, and machine-readable framework. This ensures that both current and novel procedures can be consistently reported, thereby facilitating registries, meta-analyses, and guideline development.

We suggest that future studies, registry fields, and guideline documents progressively adopt this nomenclature. Our pilot supports its feasibility and underscores its potential to unify reporting standards in hernia surgery. Ongoing projects will further explore its reliability, scalability, and integration into research and clinical practice, ultimately consolidating its role as a reference standard.

Sincerely,

Francesco Brucchi, MD

University of Milan

## Data Availability

The original contributions presented in the study are included in the article/supplementary material, further inquiries can be directed to the corresponding author.
